# Supplementary material for: An automatic weighing device for measuring the consumption of cleaning agents in mechanical cleaning equipment
Source: PLoS One. 2026 Apr 10;21(4):e0341030. doi: 10.1371/journal.pone.0341030 (PMC13068322; doi:10.1371/journal.pone.0341030)
Supplement: Attachment — Submitted filename: Response to Reviewers.docx [file pone.0341030.s003.docx]

Response to Reviewers

Dear Reviewers,

We sincerely thank you for your thorough review and for the insightful comments and suggestions you provided. Your feedback has been invaluable in helping us identify areas for improvement and in strengthening the clarity, rigor, and overall quality of our manuscript. We have carefully considered each point raised and have revised the manuscript accordingly. Below, we provide a detailed response to every comment, specifying the exact changes made.

Reviewer #1:

1. Add statistical indicators of the variability of the results. In the Results section, you should specify not only the average consumption values, but also the standard deviation or other indicator of the spread for each type of detergent. For example, “The average consumption of alkaline agent was 49.45 g ± X g (standard deviation), range Y–Z g". This will allow readers to evaluate the stability of the process over the cycles. In addition, similar figures can be given for enzyme detergent. Such data can be taken from the attached file with the original measurements. This step will make the presentation of the results more complete and quantitative.

Response: We have clearly specified the expression format of cleaning agent consumption in the Results section as "mean value + 95% CI". Specifically, this includes the data for alkaline cleaning agent (49.45 g, 95% CI: 48.51–50.40) and enzymatic cleaning agent (88.46 g, 95% CI: 85.81–91.18) when the peristaltic pump hose was not replaced, as well as the corresponding data for the two types of cleaning agents after hose replacement. The 95% confidence interval can clearly reflect the degree of data dispersion and the stability of results, helping readers evaluate the fluctuation of consumption during different cleaning cycles. This also achieves the goal of presenting results quantitatively and makes the expression of results more comprehensive.

2. To clarify the accuracy and validation of the measurements of the device. It is recommended to add a small explanation about the accuracy of the device to the Materials and Methods or Discussion section. For example, you can specify that the device is calibrated with an accuracy of ± 0.02% (based on the sensor characteristics), and therefore the measured deviations of 12-18% significantly exceed the error, which confirms the reality of the problem of underfilling. It is also advisable to describe whether the comparison was carried out with the control measurement: if the authors checked at least several cycles manually (by weighing the canister on a laboratory scale, or by measuring the volume of the pumped solution with a measuring cylinder), it is worth mentioning. If not, it can be noted that the credibility of the data is based on the sensor specifications and successful calibration with a 100 g load. This will dispel possible doubts among readers about the reliability of the testimony.

Response: To clarify the accuracy and validation of the device's measurements, we have added a device accuracy verification experiment: the washer-disinfector was started and run for 10 cleaning cycles, with the volumetric measurement method (gold standard) used as a control to compare the difference between the cleaning agent consumption calculated by the automatic weighing device and the result obtained via the volumetric measurement method. The results show that the average value of the difference between the two methods is 0.16 ml (95% CI: -0.24–0.56), and the interquartile range of the absolute value of the difference is 0.54–1.06 ml, indicating that the difference is extremely small and within an acceptable range.

3. Increase the clarity of presentation and simplify complex sentences. Check the text for excessively long and ponderous phrases, especially in the Discussion section. Try to break them down into shorter ones. For example, the sentence on pages 225-234 can be made less cumbersome. In its current form, it is quite difficult to read, although the essence is clear. Try to use neutral language and avoid rare idioms so that foreign readers can clearly understand the idea.

Response: We have optimized the sentences in the manuscript, with special attention to the Discussion section. The specific revisions are as follows: overly long sentences have been split—sentences containing multiple layers of logic are divided into shorter ones that focus on a single logical point, and simple conjunctions such as "thus/however" are used for connection to clarify the primary-secondary relationship; redundant expressions have been simplified, with repetitive statements removed and relevant expressions optimized into more common ones; uncommon vocabulary has been replaced to ensure the language is easy to understand; and logical stratification has been strengthened to avoid logical confusion.

4. Reflect the limitations of the device more explicitly and suggest solutions. Discussion already has a subsection Limits and future work, where it is noted that one device serves only one reagent, which is why duplicates of devices are needed for 2-3 reagents. It would be good to emphasize this point and, perhaps, discuss how this affects practical implementation. For example, you can mention: “In its current form, to control two reagents, it was necessary to install two copies of the device, as we did in the experiment.” Then add that in the future it is planned to combine several sensors into one system. Also mention that the application only displays data so far, and there is no way to store/analyze it through it, but this is planned to be finalized. These points are already in the text, but make sure they are noticeable enough. Perhaps it is worth concluding that the next steps will be: “creating a multi-reagent version of the device and developing the software.” This will show readers and reviewers that the authors are aware of the limits of the device's applicability and are actively working to overcome them.

Response: We have further clarified and detailed the device's limitations and improvement directions in the revised manuscript: The original manuscript mentioned that a single device can only monitor the consumption of one type of cleaning agent, and the revised version supplements the impact of this issue on practical application—multiple devices are required for the same washer-disinfector, which is prone to resource waste and increases the difficulty of management and maintenance in the Central Sterile Supply Department (CSSD). Meanwhile, we have emphasized the future improvement plan, i.e., integrating hardware and software to develop a "single infrared sensor + single display screen + multiple weighing platforms" system for simultaneous monitoring of multiple reagents.

5. Minor language and design changes. Add spaces before the brackets when entering abbreviations (PCB, GPIO, etc.). Check the uniformity of the design of the units (it should be “25 L”, “520 L”, “83 ml” with a space between the number and the unit). Correct typos, if any (for example, on pages 202-204). Reconsider the term “compliance rate” – you can rename it in the text to “percentage of expected dosage” or “delivery efficiency”, since compliance is usually used for patients, not for pumps. But if you decide to keep it, at least explain what it is when you first use it. Make sure that references to literature are formatted correctly (in the PDF text, some links are adjacent to the word without a space).

Response: We have made comprehensive revisions to the manuscript as required, with the specific changes as follows: added spaces before abbreviations (e.g., PCB, GPIO); standardized the format of units to ensure a space is retained between numbers and units (e.g., "25 L", "520 L"); checked and corrected any potential typos throughout the text; changed "compliance rate" to "dosing accuracy" to avoid confusion with the term "patient compliance"; and standardized the format of references. All revisions comply with academic writing standards, enhancing the rigor and readability of the manuscript.

6. Additional discussion of the result context. Perhaps it is worth adding a couple of sentences to the Discussion with an interpretation – what is the threat of a detected 15% shortage of detergent? The authors mentioned that this may affect the quality of cleaning and sterilization. We can give a small estimate: for example, the lack of an enzyme preparation of 12% (88 g instead of 100 g) – how critical is this? Are there studies or standards saying that even such a reduction can lead to poor-quality cleaning? Your sources [19, 20] probably indicate that the lack of detergent worsens sterilization. You could also refer here in the discussion to emphasize the importance. This would reinforce the conclusion: “Our device revealed a significant (15%) under-dose, which is potentially dangerous for the quality of the treatment, as noted in [19, 20].” Such a combination of the result with the practical consequence will make the article more convincing for the practical reader.

Response: Regarding the impact of insufficient cleaning agent delivery volume, we need to supplement the explanation based on practical application scenarios: Currently, the recommended concentration range for mainstream cleaning agents in their instruction manuals is relatively wide (e.g., 1:200–1:800). If a high-concentration ratio (e.g., 1:200) is initially set, even if the delivery volume decreases by 50%, the actual ratio (1:400) may still fall within the range permitted by the manual. It is thus difficult to directly define the absolute risk of a "15% shortage" solely based on numerical values. However, in clinical practice, the CSSD determines a fixed concentration ratio (e.g., 1:300 or 1:400) according to equipment performance, degree of instrument contamination, and other factors. This fixed ratio is a key parameter for ensuring cleaning quality and has been verified through long-term clinical practice. Strict adherence to this concentration ratio is required—once the actual delivery volume is significantly lower than this fixed value, even if it still lies within the broad range specified in the manual, it may disrupt the verified balance of cleaning effectiveness.

Additionally, there are limitations in the evaluation of cleaning effectiveness: Currently, the assessment mainly relies on macroscopic methods such as visual inspection, which makes it difficult to accurately identify trace organic matter and microbial residues. Even if insufficient delivery volume leads to incomplete cleaning, it is hard to directly attribute this issue to dosage deviation. Based on the above practical circumstances, we did not simply quantify the specific risks of a "15% shortage." However, in the Discussion section, we have emphasized that "it is necessary to monitor the consistency between the actual dosage and the fixed ratio using the automatic weighing device" and cited the core conclusion from references [23, 24] that "insufficient cleaning agent dosage affects sterilization quality." This illustrates the importance of continuous dosage monitoring and avoiding deviations from the preset ratio, thereby demonstrating the practical value of our device in ensuring the stability of clinical cleaning processes.

7. Consider storing data in a repository. In addition to the attached Excel file, consider putting the data in a public repository. This is not strictly necessary, but it is welcome and can add value to the article. If you decide to do this, specify the link in the Data Availability Statement. But even without that, make sure that the final article explicitly lists additional files with clear descriptions.

Response: Thank you for your suggestion. As requested, we have uploaded all the original data of the study to the public data repository *figshare* and obtained a permanent access link.

Reviewer #2:

1.The study presents average consumption values but lacks comprehensive statistical analysis. There is no mention of standard deviation, confidence intervals, or statistical tests to validate the significance of the findings. The authors should provide a more detailed statistical evaluation of the data collected over 52 and 50 cleaning cycles.

Response: We have clearly specified the expression format of cleaning agent consumption in the Results section as "mean value + 95% CI". Specifically, this includes the data for alkaline cleaning agent (49.45 g, 95% CI: 48.51–50.40) and enzymatic cleaning agent (88.46 g, 95% CI: 85.81–91.18) when the peristaltic pump hose was not replaced, as well as the corresponding data for the two types of cleaning agents after hose replacement. The 95% confidence interval can clearly reflect the degree of data dispersion and the stability of results, helping readers evaluate the fluctuation of consumption during different cleaning cycles. This also achieves the goal of presenting results quantitatively and makes the expression of results more comprehensive.

2.The research is based on data from a single washer-disinfector unit operating for a relatively short period. A broader study involving multiple units and longer-term observations would strengthen the validity of the conclusions.

Response: While only data from the short-term application of the device on one washer-disinfector was reported in the initial manuscript, by the time of the manuscript's major revision, the device had been tested on three different washer-disinfectors, with a cumulative operating time of 4 months. During this period, the device did not experience any failures such as software crashes or component damage. This not only verifies its adaptability to different devices but also proves the stability and reliability of the device's operation.

3.The manuscript does not provide sufficient details about the calibration procedures for the load cell and other measurement components. Proper calibration protocols and their documentation are crucial for ensuring measurement accuracy.

Response: We have added verification of the automatic weighing device's accuracy: the washer-disinfector was operated for 10 cleaning cycles, and the difference between the cleaning agent consumption calculated by the automatic weighing device and the results of the volumetric measurement method (gold standard) was compared. The test results showed minimal difference, indicating that the device's accuracy is acceptable. Relevant content has been supplemented in the manuscript. Additionally, we have clearly specified that the device is calibrated using a 100g weight, and combined with the inherent accuracy of ±0.02% of the weight sensor, this further ensures the credibility of the measurement results.

4.The study does not present data on the long-term reliability and stability of the device. Information on how the device performs over extended periods under varying conditions would be valuable for assessing its practical applicability.

Response: The initial manuscript only presented data from the short-term application of the device on one washer-disinfector. However, by the stage of the manuscript's major revision, the device has been extended to testing on three washer-disinfectors of different specifications, with a cumulative operating time of up to 4 months. No issues such as software crashes or component damage occurred throughout the entire period, which not only verifies its adaptability to various types of equipment but also further confirms the stability and reliability of the device's operation.

5.While the device uses a TF card for data storage, there is no discussion about data security, backup procedures, or protection against data loss. These aspects are critical for a device used in a healthcare setting.

Response: Currently, the device indeed only realizes local data storage via a TF card, which has limitations such as the lack of a data backup mechanism and insufficient operational convenience. We have addressed this issue in the revised manuscript. Moving forward, we will enable real-time data synchronization between the device and a mobile APP through a WiFi module. On one hand, we can rely on the APP's cloud storage function to establish dual data backups, effectively avoiding the risk of data loss caused by single-mode storage; on the other hand, this will also improve the convenience and timeliness of data acquisition, enhancing the safety and practicality of the device in medical scenario applications.

6.The cost of $65 mentioned for the device does not include installation, maintenance, or potential replacement costs of components. A more comprehensive cost-benefit analysis would provide a clearer picture of the device’s economic viability.

Response: The $65 mentioned in the manuscript represents the core hardware cost of the device. In the revised manuscript, we have supplemented the detailed price breakdown of specific modules, including a weight sensor ($32), an infrared sensor ($10), a display screen ($10), a 3D-printed box ($7), an ESP32 development board ($5), and an HX711 module ($1), which clearly breaks down the cost composition. The device adopts a modular design with a simple overall structure. This allows CSSD technicians to easily complete the installation without incurring additional installation costs, and also facilitates the replacement of individual damaged modules. For daily maintenance, only regular weight calibration using a 100g standard weight is required, resulting in essentially no maintenance costs.

7.The infrared sensor’s accuracy and reliability are not thoroughly validated. The manuscript should include more detailed information about the sensor’s performance under different conditions and potential sources of error.

Response: In this device, the core function of the infrared sensor is to determine whether the cleaning cycle has ended by detecting the "open/closed state" of the washer-disinfector's unloading door (door opening means the cycle is judged to terminate, triggering the calculation of cleaning agent consumption). Its functional positioning is a "binary switch-type judgment" (only required to identify two states: "door open/door closed"), rather than a high-precision metering application.

From the perspective of technical characteristics, this infrared sensor adopts a mature diffuse reflection detection principle. It has a simple structure and is widely used in industrial scenarios. Its identification logic is stable for fixed scenarios such as washer-disinfectors, without the need for complex environmental adaptation. At the same time, it has strong anti-interference capabilities against light and dust in the air and will not be triggered by mistake. Therefore, there is no need to conduct additional complex multi-condition performance verification.

In addition, the multiple sets of cleaning agent consumption tests reported in the manuscript all rely on the infrared sensor to accurately trigger the cycle termination signal. If there is a reliability issue with the sensor, it will directly lead to deviations in the timing of consumption calculation, and thus result in data anomalies. However, all data in the actual tests were stable and consistent, which indirectly verifies the operational reliability of the infrared sensor in long-term use.

8.The mobile app’s capabilities are mentioned briefly, but there is no detailed description of its functionality, security features, or user interface design. A more comprehensive discussion of the software component is necessary.

Response: At the current stage of this research, the mobile App is positioned as a "synchronous viewing tool for device data" with a relatively simple functional design. It only enables real-time synchronization with the content displayed on the device's local screen, and functions such as data storage, analysis, or advanced interaction have not been developed yet. This limitation has been clearly specified in the "Limitations and Future Work" section of the revised manuscript, where a subsequent function upgrade plan has also been outlined—including the addition of cloud-based data storage and historical data trend analysis. We have supplemented the app's interface images in the revised manuscript to intuitively demonstrate its user interaction logic.

9.All conclusions should be supported by the corresponding data obtained.

Response: We believe that the "conclusions" in the requirement "All conclusions should be supported by the corresponding measured data" should cover the viewpoints in both the Discussion and Conclusion sections of the manuscript—including not only conclusions related to the device’s performance and effectiveness, but also judgments regarding its practicality. We have accordingly made targeted optimizations to the manuscript, as follows:

(1) Conclusions on performance: Supplemented with quantitative data for verification

To validate the conclusion that "the device provides accurate measurements," 10 additional comparative experiments of cleaning cycles were conducted. Detailed data on cleaning agent consumption, measured by both the automatic weighing device and the volumetric measurement method (gold standard), were recorded and presented in the form of a table, providing clear numerical support for the conclusion about the device’s accuracy. To strengthen the conclusion on "the device’s adaptability and stability," supplementary information was added: the automatic weighing device has operated stably on 3 washer-disinfectors for 4 months, with no faults such as software crashes or component damage occurring during this period, demonstrating good reliability.

(2) Conclusions on practicality: Supported by observable facts or design logic

For conclusions like "easy to maintain," which are difficult to support with traditional quantitative data, evidence was provided based on observable facts or design logic. For example, the device only requires "calibration with a 100g standard weight" for maintenance, and its modular design facilitates the replacement of individual faulty modules.

(3) Optimized the logical connection between the Discussion and Conclusion sections

The structure of the Discussion section was adjusted to ensure that each viewpoint corresponds to the preceding data or factual evidence. The Conclusion section refines core conclusions based on the supplemented comprehensive information and removes generalized statements without support, ensuring that all judgments in both the Discussion and Conclusion sections have clear bases.

10.references number is critically low and reflect poor work on literature review and discussion of the results obtained.

Response: We have optimized the manuscript to address the issues of insufficient references and a weak literature review. The revisions mainly include modifying the Introduction, Background, and Discussion sections, as well as supplementing multiple high-quality references. These newly added references cover key content such as the challenges and current status of cleaning agent consumption monitoring for washer-disinfectors, and comparisons of consumption detection methods.
