# Supplementary material for: An automatic weighing device for measuring the consumption of cleaning agents in mechanical cleaning equipment
Source: PLoS One. 2026 Apr 10;21(4):e0341030. doi: 10.1371/journal.pone.0341030 (PMC13068322; doi:10.1371/journal.pone.0341030)
Supplement: Attachment — Submitted filename: Response_to_Reviewers_auresp_2.docx [file pone.0341030.s004.docx]

Response to Reviewers

Dear Reviewers,

We sincerely appreciate your professional and constructive comments on our research manuscript, which have provided important guidance for us to improve the manuscript quality. We have carefully read and fully considered all your comments, and implemented the relevant revisions and explanations one by one. We now report the detailed revision status and responses to you as follows.

Reviewer 3:

Comment 1: The manuscript primarily compares detergent consumption against expected values but does not demonstrate an actual impact on patient safety. While reduced detergent dosing is discussed, no objective evidence is provided linking the observed deviations to impaired cleaning efficacy, residual soil, microbial burden, or compromised sterilization outcomes. As a result, the clinical significance of the findings remains unclear.

Response: It is indeed a practical challenge to quantitatively and directly measure the adverse impact of reduced detergent dosage on patient safety and to obtain objective evidence linking dosage deviation to impaired cleaning efficacy, residual soil, or microbial burden. This difficulty stems from multiple key practical and technical limitations in the daily work of the Central Sterile Supply Department (CSSD), which are detailed as follows:

1. Inherent technical limitations of mainstream cleaning efficacy evaluation methods

At present, the commonly used methods for evaluating the cleaning efficacy of medical devices (including visual inspection, ATP bioluminescence detection, and residual protein testing) have obvious technical deficiencies in identifying mild to moderate cleaning defects caused by insufficient detergent dosage. For mild residual oil stains, organic contaminants, or blood stains on the surface and lumens of medical devices, these methods often fail to achieve sensitive and accurate identification—luminal contaminants are particularly difficult to detect due to structural inaccessibility. In addition, most detection methods can only identify one or two types of specific contaminants, lacking the ability for comprehensive contamination assessment, and it is thus hard to form quantifiable and reproducible objective data for mild cleaning defects.

2. Practical constraints of CSSD daily workflow

CSSD technicians face heavy daily work burdens, with limited time allocated for cleaning quality inspection of medical devices. This time shortage further reduces the possibility of accurately identifying mild cleaning defects caused by insufficient detergent dosage in actual work.

3. Potential secondary contamination from detection operations

Many cleaning efficacy testing methods require direct contact with the surface or lumens of medical devices during operation. This contact may cause potential secondary contamination of the devices that have already been cleaned, and the contaminated devices need to be returned for re-cleaning and disinfection, which not only increases the workload of CSSD but also makes technicians more cautious in conducting comprehensive testing in actual work.

4. Compensation effect of subsequent standardized sterilization procedures

All medical devices reprocessed in CSSD undergo strict standardized sterilization procedures (e.g., low-temperature hydrogen peroxide plasma sterilization, ethylene oxide sterilization, saturated steam sterilization) after cleaning. These sterilization methods can effectively eliminate microbial contamination on the device surface, even if there are minor residual stains caused by insufficient cleaning. For example, a published study has reported that hair residues in the sterile packaging of instrument trays can be completely sterilized through standard sterilization procedures and will not cause nosocomial infections in patients, but surgeons and operating room nurses all consider such contaminated sterile packages unqualified and not suitable for clinical use. Therefore, it is impossible to observe the adverse consequences of mild cleaning defects on the sterility of medical devices in clinical practice, and it is further difficult to directly link detergent dosage deviation to patient safety risks such as nosocomial infections.

Notably, the absence of directly measurable microbial safety risks does not mean that insufficient detergent dosage and potential cleaning defects can be ignored. Although residual contaminants (stains, oil stains, foreign bodies, or tissue residues) on medical devices are sterile after sterilization and will not cause infectious complications, they can still trigger local tissue hyperplasia or rejection reactions in patients during clinical use, which directly affects the clinical application effect of medical devices and the comfort of patients. In addition, residual organic/inorganic contaminants on the device surface can form a protective barrier for microorganisms in the long term, which may reduce the efficacy of sterilization and bring hidden microbiological safety risks to clinical use. More importantly, the automatic weighing device developed in this study can not only monitor the gradual reduction of detergent dosage caused by peristaltic pump hose aging but also timely detect sudden dosage abnormalities caused by hose breakage, pipeline kinking/compression, and insufficient standby detergent, which can effectively avoid severe cleaning failures of medical devices caused by extreme dosage deficiency and block potential clinical risks at the source. This is the core clinical value of the device in the daily management of CSSD.

Comment 2: It is also not evident whether the washer-disinfectors evaluated in this study were validated according to the ISO 15883 series. ISO 15883 defines the accepted framework for washer-disinfector compliance, including dosing reproducibility and cleaning performance verification. Without confirmation that the tested equipment was properly validated (IQ/OQ/PQ), it is difficult to determine whether the observed dosing variability reflects a limitation of current standards or deficiencies related to equipment condition, maintenance, or validation practices.

Response: We thank the reviewer for pointing out this key point related to international standard compliance. The washer-disinfectors involved in this study were all strictly validated and qualified in accordance with the ISO 15883 series of standards before clinical use, and complete and standardized installation qualification (IQ), operational qualification (OQ), and performance qualification (PQ) documents have been retained and archived. The daily use, maintenance and performance verification of the equipment also strictly follow the requirements of ISO 15883 and the manufacturer’s operating specifications.

The detergent dosing variability observed in our study is not caused by the non-compliance of the equipment itself with ISO 15883 standards, nor by defects in the initial IQ/OQ/PQ validation process, but by the inevitable aging and wear of peristaltic pump hoses during long-term clinical use. As a core consumable part of the washer-disinfector’s dosing system, the peristaltic pump hose will gradually lose its elasticity after repeated mechanical compression, and its cross-section will change from a circle to an ellipse, resulting in a gradual decrease in the actual delivery volume of detergent per cycle. This is a common and universal problem in the daily operation of mechanical cleaning equipment that has passed ISO 15883 validation, and the current industry lacks clear and unified authoritative guidelines for the replacement cycle and performance evaluation standards of peristaltic pump hoses. Our study just targets this practical pain point in the post-validation use stage of ISO 15883-compliant equipment and provides a simple, low-cost and high-precision technical solution for real-time and accurate monitoring of detergent dosing variability.

In addition, ISO 15883 clearly stipulates that the dosing system of washer-disinfectors must ensure the accuracy and reproducibility of detergent dosage in each cleaning cycle, which is the core requirement for the normal operation of the equipment. The automatic weighing device developed in this study is highly consistent with this core requirement of ISO 15883: it can realize real-time monitoring, automatic calculation, and abnormal early warning of detergent consumption for each cleaning cycle, help CSSD technicians timely correct dosage deviations and maintain the stable and qualified dosing performance of the equipment, and thus serve as a practical and effective technical supplement to the ISO 15883 standard in the daily management and performance maintenance of mechanical cleaning equipment.

Comment 3: Importantly, medical devices reprocessed in hospitals are also validated by their manufacturers using hospital washer-disinfectors as part of regulatory submissions. Within the FDA’s total product life cycle framework, device reprocessing performance is continuously monitored post-market. At present, there are no publicly available FDA concerns indicating a systemic patient safety risk related to detergent dosing variability in validated washer-disinfector systems.

Response: We fully agree with the reviewer’s statement about the FDA’s regulatory status and the manufacturer’s validation requirements for medical device reprocessing, and we further supplement and explain the research results and their scientific significance in combination with the actual clinical practice of CSSD:

1. Detergent dosing variability in ISO 15883 validated systems has been fully confirmed in this study

Our study provides direct and objective experimental data to prove that detergent dosing variability caused by consumable wear is a persistent problem in the clinical use of ISO 15883 validated washer-disinfectors. The tested washer-disinfector, which had passed ISO 15883 validation and completed 4,426 cleaning cycles in clinical use, showed significant detergent dosage deviation: the average consumption of enzymatic detergent and alkaline detergent in 52 cleaning cycles was 88.46 g and 49.45 g, respectively, which were significantly lower than the expected values (100 g and 60 g) determined based on ISO 15883 dosing requirements, cleaning agent instructions and actual equipment water consumption. After replacing the aged peristaltic pump hoses, the average detergent consumption recovered to 97.70 g (enzymatic) and 59.67 g (alkaline), which were close to the expected values. This set of data clearly verifies that dosing variability is a common practical problem in the post-validation stage of ISO 15883-compliant equipment, independent of the FDA’s current public regulatory concerns.

2. The difficulty of measuring cleaning/patient risks caused by mild dosage variability is a widespread technical bottleneck in the current field, not a deficiency of this study

As explained in the response to the first comment, it is currently difficult to obtain measurable and reproducible objective data on the cleaning defects and patient safety risks caused by mild to moderate detergent dosage deviation. This difficulty is due to the inherent technical limitations of mainstream cleaning efficacy detection methods, the practical workflow constraints of CSSD, the compensation effect of subsequent sterilization procedures, and the lack of sensitive evaluation indicators for non-infectious clinical risks (e.g., tissue hyperplasia/rejection caused by sterile residual contaminants). This is a widespread technical bottleneck in the field of medical device reprocessing at present, which cannot be solved by a single study and requires joint efforts and in-depth research by the entire industry.

Our study does not aim to directly prove the clinical harm of mild detergent dosage variability, but to solve the long-standing practical problem of “unable to timely and accurately monitor detergent dosage variability” that plagues CSSD technicians in the daily management of ISO 15883 validated equipment. The core scientific and practical value of this study is to develop a novel automatic weighing device based on embedded technology and gravimetric method, which has the advantages of reliable measurement performance, simple structure, high compatibility, low cost and easy operation. This device can realize real-time, automatic and accurate monitoring and recording of the consumption of different cleaning agents in each cleaning cycle for ISO 15883 validated washer-disinfectors, filling the technical gap of post-validation dosing monitoring of mechanical cleaning equipment in CSSD. At the same time, the device provides a reliable technical tool for the follow-up in-depth research in the field (e.g., the correlation between long-term mild detergent dosage deviation and cleaning efficacy/sterilization effect, the establishment of sensitive evaluation indicators for non-infectious clinical risks caused by sterile residual contaminants), and lays a solid technical foundation for solving the current industry technical bottleneck of “measuring cleaning/patient risks caused by dosage variability”.

In summary, our study targets the practical pain points in the daily management of CSSD and the post-validation use of ISO 15883 compliant mechanical cleaning equipment, develops a novel automatic weighing device with independent technical characteristics, and the research results and conclusions have clear scientific significance and important clinical application value for the refined management of cleaning agent use, the performance maintenance of dosing systems, and the quality assurance of medical device reprocessing in CSSD.

We have fully considered the reviewer's comments, and the above explanations are to further clarify the research background, practical value, and the current technical limitations of the field, without changing the original research content and conclusions of the manuscript.

Reviewer 4:

Comment 1: Technical Validity and Research Conclusions

The device design (ESP32 + HX711 + load cell + infrared trigger + local display/storage and Wi-Fi application) and the operating logic are suitable for the specified objective. The field dataset, comprising 52 alkaline cycles and 50 enzymatic cycles after the exclusion of two anomalous events, substantiates the primary conclusion that the device is capable of recording per-cycle consumption and detecting systematic under-consumption in relation to the anticipated targets. Nevertheless, the manuscript should moderate or validate the assertion of "accuracy." Although sensor specifications indicate high precision, it would be advantageous for readers to have a concise validation against an external reference (e.g., a calibrated scale or a selection of cycles with independent gravimetric assessments), and/or to report fundamental measurement performance metrics (repeatability, drift, recalibration frequency).

Response: Thank you for your recognition of the rationality of the device design and the reliability of the core conclusions of this study. Regarding the suggestions on the verification of the device's measurement accuracy, this study has fully ensured the accuracy and credibility of the measurement results through three rigorous verification steps, and thus no adjustments have been made to the relevant statements in the original manuscript. The specific explanations are as follows:

1. The load cell selected in this study is a factory-qualified product, which has undergone professional precision calibration ex-factory and is accompanied by a qualification certificate, providing authoritative ex-factory verification guarantee for its basic measurement performance;

2. During the actual use of the device, we strictly implement a standardized weight calibration operation, completing zero calibration and 100 g weight calibration before each startup to ensure the accuracy of the measurement baseline. This calibration process has been described in detail in the *Materials and Methods* section of the original manuscript;

3. We conducted 20 sets of comparative tests (10 sets each for enzymatic detergent and alkaline detergent), comparing the measurement results of this device with the volumetric measurement method (the gold standard). The results showed that the mean difference in measurements was only 0.16 mL (95% CI: −0.24 to 0.56), and the interquartile range of absolute differences was within the acceptable range of 0.54 mL to 1.06 mL, which directly verified the high consistency between the device's measurement results and the gold standard.

Based on the above three verifications, we believe that the demonstration of the device's measurement accuracy in the manuscript is sufficiently adequate and rigorous, so no modifications have been made to the relevant statements. We hope for your understanding.

Comment 2: Statistical Analysis

Considering the study's descriptive and feasibility objectives, the present use of descriptive summaries and visual representations is appropriate. However, rigour and interpretability might be enhanced by presenting variability (e.g., standard deviation, median, interquartile range) and, if possible, straightforward confidence intervals surrounding mean consumption. Should the authors suspect ageing of the pump or tubing, a concise exploratory examination of temporal trends (e.g., run order vs consumption) may provide valuable insights; nevertheless, this is optional and not requisite for acceptance.

Response:

1. Supplement and Improvement of Statistical Indicators

In response to your suggestion of supplementing variability descriptors, we have improved the textual description in the *Results* section of the original manuscript. On the basis of the original 95% confidence intervals, we have added statistical indicators such as standard deviation, median and interquartile range for the consumption of enzymatic detergent and alkaline detergent before and after hose replacement. The addition of these indicators makes the distribution characteristics of the experimental data clearer and more intuitive, further enhancing the rigor and interpretability of the research results.

2. Explanation of Exploratory Analysis on Temporal Trends of Cleaning Cycles and Consumption

Regarding the exploratory analysis on the temporal trend between cleaning cycle order and cleaning agent consumption, we did not conduct this analysis after comprehensive consideration, and we apologize for not explaining this in the original manuscript. The aging rate of peristaltic pump hoses is comprehensively affected by multiple non-standardized factors in clinical practical application, including the hose materials of different washer-disinfectors, the daily usage frequency of equipment in various Central Sterile Supply Departments (CSSD), the types of cleaning agents used, and the contamination level of medical devices to be cleaned. These factors show significant heterogeneity in different application scenarios, leading to the lack of a unified law in the temporal trend of cleaning agent consumption. Even if this trend exploration is carried out based on the data of this study, the obtained results can only reflect the characteristics under the specific experimental conditions of this study, and cannot provide universally guiding significance for the clinical practice of other CSSDs. Therefore, this exploratory analysis was not conducted in this study. We have added this explanation in the *Discussion* section of the revised manuscript for readers' reference.

Comment 3: Generalisability and Limitations

The restrictions section is suitable but could be elaborated to clarify generalisability beyond a single site and a specific washer-disinfector. Additionally, please address potential sources of measurement inaccuracy in practical applications, including container displacement, vibrations, uneven flooring, refilling during operation, and environmental factors. The omission of the two enzymatic cycles resulting from refilling during pump operation is justifiable; kindly furnish a definitive operational recommendation to prevent this situation and to maintain data integrity.

Response:

1.Explanation of the Device's Generalisability

Thank you for your attention to the generalisability of the device. The *Discussion* section of the original manuscript has elaborated on the good generalisability of the device in detail: the device adopts a modular design and is completely independent of the main structure of mechanical cleaning equipment. No modification to the original pipelines and control systems of the equipment is required during installation, which greatly simplifies the installation process and will not interfere with the normal operation of the equipment. Meanwhile, the infrared sensor of the device has strong anti-interference ability and flexible installation position, which can be stably applied to washer-disinfectors with different door structures (vertical-lift, side-hinged, glass and metal doors). It is not only applicable to the specific equipment in this study, but also can be popularized to various types of mechanical cleaning equipment in the vast majority of clinical CSSDs, with a good clinical application prospect.

2.Supplementary Specifications for Avoiding Measurement Errors in Practical Application

In response to your concern about the potential sources of measurement errors in practical application, we have added two categories of standardized operation specifications for avoiding measurement errors in the *Discussion* section of the revised manuscript, providing clear guidance for clinical practical use:

2.1 Hardware placement specifications (addressing container displacement, vibration, uneven flooring, etc.): The load cell of the device must be fixed on a flat and hard ground, and the cleaning agent reservoir must be stably placed at the center of the load cell and shall not be moved at will during the operation of the equipment. The vibration generated by the operation of the washer-disinfector is transmitted to the load cell through the ground, and its intensity is significantly weakened after transmission, with a negligible impact on the measurement results and no influence on the measurement accuracy of the device.

2.2 Cleaning agent refilling specifications (addressing refilling during operation): Refilling of cleaning agents shall be completed before the daily startup or after the end of daily work of the washer-disinfector. Before the daily startup, it is necessary to check and ensure that the amount of cleaning agent in the reservoir is sufficient for the day's use. If refilling is really necessary during clinical work, it must be carried out in the interval after the end of the previous cleaning program and before the start of the next one. Refilling during the operation of the cleaning program is strictly prohibited to avoid distortion of measurement data.

We believe that the strict implementation of the above standardized operation specifications can effectively avoid potential measurement errors in practical application and ensure the accuracy and reliability of the device's measurement results.

Comment 4: Accessibility of Data and Research Reproducibility

The Data Availability Statement specifies that all pertinent data are contained within the paper and Supporting Information files, with additional data/code provided. Kindly guarantee that the data file is thoroughly documented (including variable names, units, cycle identifiers, and any missing-data indicators) to enable readers to replicate Figure 4 and the stated means/compliance rates unequivocally.

Response: Thank you for your suggestions on improving data documentation. All experimental data of this study have been uploaded to a public database, and the relevant data files are standardized in annotation and complete in content: all detection indicators have clear variable names and unified measurement units, with no missing or abnormal data in the dataset. In addition, the calibration test data corresponding to Figure 4 and all statistical results (such as mean values and compliance rates) stated in the manuscript are clearly marked and correlated in the data files. Readers can directly and unequivocally reproduce Figure 4 and calculate the stated mean values, compliance rates and other statistical results through the uploaded experimental data. Meanwhile, the experimental methods and operation steps of this study are described in detail in the manuscript, and other researchers can repeat the experiment according to the methods in the manuscript to obtain similar experimental results, which fully ensures the reproducibility of this study.

Comment 5: Language and Presentation Format

The manuscript is coherent and predominantly composed in standard scientific English. It is advisable to undertake some linguistic refinement, especially in the Discussion and Conclusion sections, to enhance fluency and conciseness. Kindly rectify minor formatting discrepancies (e.g., insert a space in “printed circuit board (PCB)”) and condense some lengthy lines.

Response:

1. We have conducted a comprehensive check on the language of the full text and revised all potential minor formatting issues (such as splitting overly long sentences and standardizing punctuation usage), making the typesetting of the manuscript clearer and more readable.

2. On the premise of ensuring the rigor of scientific expression, we have carefully polished the language of the *Discussion* and *Conclusion* sections of the manuscript, streamlined redundant expressions, optimized the logical connection between sentences, and improved the fluency and conciseness of the text.

We sincerely thank you again for your valuable comments and professional guidance, which have significantly improved the quality of this manuscript. We anticipate that the revised manuscript meets the publication requirements and welcome your further guidance.
