# Supplementary material for: An automatic weighing device for measuring the consumption of cleaning agents in mechanical cleaning equipment
Source: PLoS One. 2026 Apr 10;21(4):e0341030. doi: 10.1371/journal.pone.0341030 (PMC13068322; doi:10.1371/journal.pone.0341030)
Supplement: Attachment — Submitted filename: Response_to_Reviewers_auresp_3.docx [file pone.0341030.s005.docx]

Response to Reviewers

Reviewer #3:

We sincerely appreciate the insightful and constructive comments from the reviewer, which have prompted us to conduct further in-depth thinking and reflection on our research. Below, we provide detailed explanations and clarifications on several key issues raised in the comments.

The cleaning performance of washer-disinfectors relies on the synergistic operation of all equipment components, water quality, and cleaning agents, among which accurate dosing of cleaning agents is an indispensable prerequisite for ensuring cleaning quality. The core objective of this study is to develop a convenient and feasible method to identify reductions in cleaning agent consumption. A decrease in cleaning agent dosage directly impairs cleaning quality in clinical practice, which constitutes the fundamental rationale for conducting this research.

In response to the first comment: the absence of laboratory-based controlled studies to verify whether reduced cleaning agent delivery impairs cleaning quality and determine the critical non-conforming ratio

We provide a detailed explanation as follows: We have not conducted such laboratory tests primarily for two reasons. First, such tests are entirely unfeasible in practice. For one thing, there is an extremely diverse range of medical devices with significant variations in structure, material, size, and volume. Moreover, medical devices used in operating rooms are processed by the Central Sterile Supply Department (CSSD) as soon as possible and then stored in operating room warehouses for standby use, making it impossible to retain all types of devices for an extended period for experimental purposes. For another, there are hundreds or even more types of cleaning agents available on the market, each with distinct compositions, formulations, and recommended usage concentrations, rendering it impractical to test every single type of cleaning agent. In addition, washer-disinfectors in different CSSDs vary in terms of manufacturer, model, and service life, coupled with differences in water quality for cleaning and the flushing pressure of rotating walls. Furthermore, quantitatively simulated blood and soil contaminants in the laboratory differ drastically from the actual contamination status of operating room devices after use, and cannot truly replicate clinical scenarios. Therefore, any conclusions drawn from such laboratory tests would lack guiding significance and be unable to be popularized or applied in clinical settings. Second, our research team lacks the conditions to conduct such laboratory tests, including the absence of a full range of medical devices dedicated to experiments and standardized simulated contamination materials.

It is particularly important to emphasize that the core focus of this study is not to identify the "specific ratio of cleaning agent reduction that leads to non-conforming cleaning outcomes", but to address the most critical clinical demand of CSSDs—ensuring that the dosage of each type of cleaning agent meets the set standard in every cleaning cycle. Peristaltic pump or hose aging inevitably causes a gradual decrease in cleaning agent delivery, which will certainly result in non-conforming cleaning quality. This is an unquestionable fact in clinical practice and a well-recognized common sense in the industry, requiring no additional verification through laboratory tests. Moreover, in extreme cases, even if the peristaltic pump is not damaged, issues such as kinking, breakage, or disconnection of the cleaning agent delivery pipeline can be promptly detected using our research method, which precisely reflects the clinical practical value of this study.

In response to the second comment: the request for evidence of the washer-disinfector’s compliance with ISO 15883 validation and relevant OQ/PQ documentation

We respond as follows: All washer-disinfectors tested in this study are manufactured by reputable European and Asian manufacturers. To obtain market access qualifications and be legally sold, these devices must have passed validation in accordance with ISO 15883 or equivalent regional standards. This is analogous to motor vehicles legally sold in any country, which must comply with local regulations and pass rigorous validation, ensuring their factory compliance. Therefore, we believe it is unnecessary to provide additional equipment validation documents.

There is no contradiction between a washer-disinfector passing ISO validation and exhibiting systematic reductions in cleaning agent delivery. Peristaltic pump hoses are consumables that require regular replacement, yet their aging rate is affected by multiple factors including pump material, service time, usage frequency, and the medium being delivered. No manufacturer can provide an accurate and fixed replacement schedule for hoses, and the core value of this study lies precisely in the early detection of abnormal aging of peristaltic pump hoses. With regard to the OQ/PQ validation of cleaning agent dosing, multiple rounds of validation have been conducted during equipment installation and replacement of new cleaning agents, including visual inspection under a magnifying glass with a light source, cleaning efficacy test card assays, and ATP testing. The same standardized tests are also performed on a weekly basis to ensure the cleaning quality of medical devices.

In addition, the contamination degree of different medical devices varies significantly in clinical practice: some devices in surgical instrument sets may remain clean without being used, some are lightly contaminated due to infrequent use, and others are heavily contaminated due to frequent use or application in highly contaminated surgical sites. Meanwhile, cleaning efficacy is also affected by factors such as contaminant desiccation, contaminant location, and preprocessing quality. Consequently, if medical devices cleaned by washer-disinfectors exhibit non-conforming cleaning quality, technicians may not first attribute the issue to peristaltic pump aging. Conversely, if insufficient cleaning agent delivery caused by peristaltic pump aging leads to cleaning failure, CSSD technicians may misjudge the cause as excessive device contamination or inadequate preprocessing, rather than examining the peristaltic pump. To reduce such cognitive biases and misattribution of responsibilities, we argue that the most essential and achievable measure is to ensure the peristaltic pump delivers the set dosage of cleaning agent in each cycle from the source, which is also the core goal of this study.

In response to the third comment: the manuscript implies safety concerns associated with significant deviations in cleaning agent delivery without citing supporting evidence from regulatory authorities such as the U.S. Food and Drug Administration (FDA)

We explain as follows: The manuscript does mention in the background section that significant reductions in cleaning agent delivery pose potential safety risks, which is a reasonable inference based on clinical logic. Persistent insufficient cleaning agent dosage directly impairs cleaning efficacy, leading to residual contaminants on medical devices and thereby increasing the risk of cross-infection—a self-evident fact in clinical practice. At present, international standards (e.g., the ISO 15883 series) and Chinese industry standards (e.g., the WS 310 series) all require that cleaning agent dosing should adhere to the principles of "controllability, adjustability, and monitorability". However, it is true that there are no specific guidance documents, safety alerts, product recalls, MAUDE reports, or official announcements issued by regulatory authorities such as the U.S. FDA regarding this issue. This absence, however, does not negate the necessity and importance of this study.

Cleaning agents are a key factor in ensuring cleaning quality. Peristaltic pump aging leads to a persistent and progressive decrease in cleaning agent delivery, which will ultimately result in non-conforming cleaning quality of medical devices. Although regulatory authorities have not yet clearly specified refined management methods for deviations in cleaning agent delivery, this study focuses on early detection of peristaltic pump hose aging to ensure consistent cleaning agent delivery dosage. It is highly aligned with the actual clinical needs of CSSDs and addresses the shortcomings of existing technologies, thus possessing significant clinical practical value.

Reviewer #4:

We sincerely appreciate your meticulous review and insightful comments on our manuscript, as well as your recognition of the revisions we have made. Your affirmation of the methodological rigor of the research, the effectiveness of the field application, and the scientific rigor of the conclusion presentation is a great encouragement to our work. We would like to express our sincere gratitude again for your professional suggestions, which have provided important support for the improvement of this manuscript.
